# Anthropometry and body composition of school children in Bahrain

**DOI:** 10.4103/0256-4947.55309

**Published:** 2009

**Authors:** Nadia M. Gharib, Parveen Rasheed

**Affiliations:** From the ^a^Nutrition Section/Public Health Directorate-Ministry of Health, Kingdom of Bahrain and ^b^Department of Family & Community Medicine, King Faisal University, Dammam, Saudi Arabia

## Abstract

**BACKGROUND AND OBJECTIVES:**

This study was conducted because of the lack of a comprehensive nationwide assessment of data on the anthropometric status and related health problems in Bahraini school children aged 6 to 18 years.

**SUBJECTS AND METHODS:**

A cross-sectional survey was conducted on the anthropometric status of school children enrolled in the primary, intermediate and secondary government schools in all populated regions of Bahrain. The sample size included 2594 students (1326 girls and 1268 boys) representing 2.5% of the total student population. For sample selection, a multi-stage sampling design was chosen that combined multi-cluster and simple random sampling methods. Anthropometric measurements included height, weight, mid-arm circumference and skin fold thickness at two sites (triceps and subscapular). Anthropometric indices derived were body mass index (BMI) and arm muscle area. The WHO reference standards (2007) and the National Health and Nutrition Examination Survey (NHANES) II data were used for comparison.

**RESULTS:**

Compared to WHO reference standards, the median height of Bahraini children and adolescents in the age range of 6 to 18 years was close to the 25th percentile or lower, while the median BMI during adolescent years was comparable in boys, but higher than WHO standards in girls, reaching the 75th percentile. The cut-off values of BMI for overweight/obesity status (85th and 95th percentile) were higher by 3-6 kg/m^2^ compared to WHO standards. While skin fold thicknesses were also higher in Bahraini adolescents compared to their American counterparts (NHANES II), arm muscularity was substantially lower.

**CONCLUSIONS:**

Current study findings for BMI as well as skin fold thicknesses suggest an increased trend toward adiposity among Bahraini adolescents, especially in girls, which puts this age group at a high risk of adult obesity and its consequences. A need for urgent intervention programs is emphasized.

Anthropometry is used to characterize growth patterns and body composition. Growth patterns are indicators of nutritional status of children and are important in developing intervention programs.^1^–^9^ Previous nutritional surveys in Bahrain were limited in their scope and sampling. Some were designed to provide more comprehensive and representative data on the growth patterns of Bahraini children.^1^–^3^ Musaiger et al in 1989 reported that the median heights and weights, triceps skin fold and median arm circumference of boys and girls aged 6.5 to 18.5 years were below the 50th percentiles of the American reference standard (National Center for Health Statistics, NCHS) (3). A decade later (2000), data from the National Growth Survey for Bahraini children showed that the mean body mass index (BMI) of girls aged ≥13 years exceeded that of their American counterparts, but the muscles of children of all ages and both sexes appeared to be underdeveloped. The authors expressed concern over a rising trend for obesity, especially in girls, and emphasized the need for intervention programs.^2^ The findings of Al-Sendi et al in 2003 further confirmed the increasing weight gain among the adolescent population.^1^ The current research was conducted on a large and representative sample of Bahraini school children and adlolescents aged 6 to 18 years from all the populated regions of the country and formed one of the components of a comprehensive assessment of nutritional status, particularly, anthropometric status (height, weight, body mass index, subscapular skin fold, triceps skinfold thickness, midarm circumference and arm muscle area).

## SUBJECTS AND METHODS

The study was cross-sectional and descriptive. Data collection was from January 1999 to May 2001. The target population was Bahraini boys and girls in primary, intermediate and secondary levels in public schools of the 11 populated regions of Bahrain. Information on children aged ≥10 years old was obtained through a self-administered questionnaire while that for younger children was completed by the parents. The age of the children was verified from school records and recorded in whole years.

### Sampling

Sample size was determined to ensure a sufficient number of subjects in each age and sex group, according to WHO criteria (1995).^10^ An estimated sample size of 2443 was obtained using the standard statistical formula designed to produce valid results for anthropometric variables so that the results are within an approximately 95% confidence interval estimate of the growth parameters. A multi-stage sampling design that combined multi-cluster and simple random sampling methods was chosen to select the sample. Cluster sampling was used in two successive stages: first, for allocation of the schools in each region in proportion to the size of the region and second, for selection of students in proportion to the number of educational levels in each school. Then, the allocated numbers of the students from each level were chosen randomly from different classes.

There were 190 government schools (94 for girls and 96 for boys) in Bahrain during the school year of 1998/1999, enrolling a total of 104189 Bahraini students (52885 girls; 51304 boys) from ages 6 to 18 years.^11^ The study included 2594 children (1326 girls, 1268 boys), which represented 2.5% of the total population of school children in Bahrain for the years 1998 to 1999. Parental consent was required for Inclusion of a student in the study. An informed consent form was sent to all the parents of the children. Almost all (99%) parents provided signed consent forms.

### Anthropometric measurements

Measurements of height, weight, mid-upper arm circumference (MUAC) and skin fold thickness were obtained for the selected children. All measurements were taken three times and an average value was recorded. Height and weight measurements were taken with the help of a digital electronic scale (SECA Model 930, Hamburg, Germany). The scales were checked for accuracy and calibrated by a specialist prior to the start of fieldwork and during the fieldwork. The technique of recording weight, height and MUAC was done according to guidelines suggested by The WHO Expert Committee (1995).^10^ A team of four nurses were trained by one of the investigators (NG) on the correct procedure for taking anthropometric measurements. Their work was supervised periodically during the survey.

Height measurements were taken without shoes. The students were positioned with their feet together and flat on the base plate with their head and back straight against the vertical measuring rods. Once the correct position was achieved the interviewer lowered the head plate until it just touched the top of the student's head, and while maintaining this position, he or she was asked to stand as tall as possible, without lifting the heels. Measurements were made to the nearest 0.1 cm. Weight measurements were taken in light clothing; shoes, trainers, jackets, heavy jewelry, keys and wallets were removed. Weight was recorded to the nearest 0.1 kg.

MUAC was measured with the student's left arm at 90° across the body. Using a conventional non-stretchable tape (metal tape from Chasmors LTD, London), the distance between the inferior border of the acromion and the tip of the olecranon process was measured and the mid-point on the student's arm was marked. The insertion tape was then placed horizontally at the level of the mid-point without compressing the tissues and a circumference measurement was taken to the nearest 0.1 cm.

Triceps skin fold (TSF) and sub-scapular skin fold (SSF) thickness were measured using a Harpenden skin fold caliper (Crymych, Wales, UK). Measurement of TSF was taken on the posterior aspect of the bare extended right arm, over the triceps muscle, midway between the lateral projection of the acromion process of the scapula and the inferior margin of the olecranon process of the ulna. The caliper tips were placed perpendicular to the long axis of the skin fold, and the reading on the dial was taken to the nearest 0.1 mm. SSF was measured 2 cm below the lowest or inferior angle of the scapula. The long axis of the skin fold was at a 45° angle directed to the right side. With the student's arms relaxed to the sides, the skin was grasped 1 cm above and medial to the site along the axis. A measurement was taken to the nearest 0.1 mm. Both measurements were taken according to the methods described by Nieman and Lee.^12^

### Anthropometric indices

Body mass index (BMI) was calculated by using the formula weight (kg) divided by height (m)squared.^10^ Cross-sectional arm muscle area (AMA) was estimated from upper arm circumference (UAC) and TSF, assuming a circular and concentric model, using the formula^10^

AMA(cm)2=[UAE-(π_TSF)]/4π

For construction of height, weight and BMI percentiles, a statistical analysis was performed using the LMS (lambda, mu, sigma) method. The calculation of the smoothed percentiles was obtained using standard software.^13^

### Reference standards

Height, weight and BMI were compared to the newly recommended NCHS/WHO reference standards (2007);^14^ MUAC and AMA were compared to data of the US National Health and Nutrition Examination Survey (NHANES) 2 of 1971-1974, which were derived from population samples of the National Center for Health Statistics (NCHS) growth percentiles for children^15^ and TSF and SSF were compared to US data adopted from the NCHS, 1987.^16^

### Pilot study

A pilot study was conducted on 60 boys and 60 girls (5% of sample) who were chosen randomly from six schools of one region. Thirty students of each gender were selected from the primary schools and 15 each from the intermediate and secondary schools. Each academic level was represented. The pilot study was done to identify any possible administrative difficulties, the accuracy of the procedures involved, time motion and the response rate of students. No modifications were required in the anthropometric component of the study.

## RESULTS

Of 2594 students in the study population, 53.4% (n=1386), 23.0% (n=596) and 23.6% (n=612) were from primary, intermediate and secondary schools, respectively. Their ages ranged from 6 to 18 years, with a mean (SD) age of 12 (3.6) years. Almost 54% (n=713) of the girls (12 to 18 years) and 48% (n=606)of the boys (13 to 18 years) were in the adolescent age group as per criteria defined by Story et al.^17^

Tables 1 and 2 show the means for the anthropometric variables by age and gender. The means of the anthropometric measurements gradually increased with age in both genders with the mean height of the girls and boys beyond the ages of 16 years and 17 years, respectively, remaining relatively unchanged. The mean MUAC and AMA for girls gradually increased with age up to 17 years, but showed a slight decrease thereafter. A similar trend by age was observed in boys for body weight, BMI and MUAC.

**Table 1:** Anthropometric measurements and indices for Bahraini girls.

Age	n	%	Height (cm)	Weight (kg)	Body mass index (kg/m^2^)	Subscapular skin fold thickness (mm)	Triceps skin fold thickness (mm)	Midarm circumference (cm)	Arm muscle area (cm)
6	50	4	118.7 (6.4)	21.1 (5.1)	14.8 (2.4)	7.1 (4)	10.5 (4.7)	13.6 (4.8)	10.6 (7.7)
7	87	7	120 (5.7)	21.6 (5.8)	14.9 (3.6)	7.2 (3.3)	10.9 (4.7)	14.4 (5.6)	12.1 (8.9)
8	90	7.3	125.4 (5.7)	24.8 (7.7)	15.5 (4.1)	8.2 (4.5)	13 (7.8)	13.6 (4.9)	9.8 (8.3)
9	119	9.6	130.3 (7.2)	28.1 (8.1)	16.4 (3.8)	9.1 (5.4)	13.7 (7.3)	15.9 (6.3)	13.9 (10.8)
10	116	9.3	136.4 (7.9)	33.9 (10.7)	17.9 (4)	10.7 (6.5)	15.4 (8.3)	16.7 (8.7)	16.8 (27.3)
11	123	9.9	141.8 (7.5)	38.3 (11.5)	18.8 (4.6)	12.3 (7.3)	16.5 (8.9)	18 (7.2)	17.2 (13.3)
12	104	8.4	147.6 (7.3)	43.7 (12)	19.9 (4.8)	12.7 (7.4)	17.8 (8.8)	18.7 (7.7)	18.1 (14.1)
13	83	6.7	152.8 (6.8)	50.9 (12.9)	21.6 (4.5)	15.3 (8.1)	22.1 (9.8)	22.8 (7.4)	23.9 (13.6)
14	97	7.8	156.1 (6.4)	54.3 (14.3)	22.2 (5.3)	16.1 (9.4)	23.4 (10)	22.2 (8.4)	22.6 (15.6)
15	71	5.7	155.5 (5.2)	54.3 (13.2)	22.4 (5)	17.2 (10.4)	23.7 (12)	24.7 (5.5)	25.4 (12)
16	108	8.7	156.6 (5.9)	56.7 (13.2)	23.1 (5.1)	19.4 (9.6)	26.7 (10.4)	25.1 (5.5)	23.9 (12.5)
17	97	7.8	156.3 (6.6)	57.6 (16.8)	23.3 (5.4)	20 (10.4)	28.9 (13)	27.3 (6.4)	28 (13.4)
18	90	7.3	156.6 (6.1)	57.2 (12.6)	23.3 (4.7)	20.2 (10.1)	29.2 (12.6)	26.9 (5.2)	26.1 (11.5)
**Total**	**1235**	**100**	**143.2 (14.9)**	**42 (17.6)**	**19.6 (5.5)**	**13.6 (9.0)**	**19.5 (11.3)**	**20 (8.2)**	**19.2 (15.4)**

Values are mean (standard deviation).

**Table 2:** Anthropometric measurements and indices for Bahraini boys.

Age	n	%	Height (cm)	Weight (kg)	Body mass index (kg/m^2^)	Subscapular skin fold thickness (mm)	Triceps skin fold thickness (mm)	Midarm circumference (cm)	Arm muscle area (cm)
6	31	2.6	118 (5.3)	20.2 (4.9)	14.3 (2.6)	5.5 (6.0)	7.2 (5.3)	17 (2.4)	16.9 (2.9)
7	89	7.5	121.5 (5.8)	21.5 (5.0)	14.6 (2.5)	5.3 (3.2)	7.5 (3.2)	17.5 (2.1)	17.7 (3.3)
8	113	9.5	126 (5.8)	23.7 (5.5)	15.2 (2.7)	6 (4.0)	7.6 (4.3)	17.8 (2.5)	19.3 (4.0)
9	98	8.2	130.5 (6.4)	27 (8.8)	15.8 (3.9)	7.1 (6.1)	8.6 (6.6)	19 (3.5)	21.3 (4.7)
10	101	8.5	134.5 (6.7)	29.7 (9.5)	16.5 (4.1)	7 (7.6)	9.7 (5.9)	20 (3.2)	22.2 (4.7)
11	110	9.3	139.5 (6.7)	33.6 (10.4)	16.6 (4.0)	7.1 (7.9)	9.2 (6.7)	20.4 (3.4)	23.5 (5.0)
12	90	7.6	146.3 (9.4)	40.6 (14.5)	18.7 (5.0)	9.8 (11.0)	11.7 (9.4)	22.6 (4.4)	27.5 (7.6)
13	106	8.9	151.3 (8.9)	42 (12.0)	18.9 (4.1)	8.8 (9.1)	11 (7.2)	23.3 (3.3)	28.5 (6.7)
14	98	8.2	162 (9.1)	51.7 (14.7)	19.5 (4.5)	9 (9.6)	10 (8.2)	24.5 (3.9)	35.3 (7.9)
15	85	7.1	164 (8.3)	53.2 (14.2)	19.7 (4.2)	8.3 (10.0)	8.7 (8.9)	24.5 (4.2)	36 (9.4)
16	86	7.2	169 (6.9)	61.7 (16.7)	21 (5.8)	9.4 (10.4)	9.8 (9.6)	27 (4.3)	43.3 (8.4)
17	90	7.6	171 (6.6)	63.2 (21.4)	21.6 (7.6)	11.7 (14.4)	11.1 (10.9)	27.8 (4.9)	47.7 (11.1)
18	76	6.4	171 (6.5)	64.9 (10.8)	21.8 (3.3)	11 (6.2)	8.8 (6.7)	28 (3.4)	47 (10.9)
**Total**	**1173**	**100**	**145 (18.9)**	**39.9 (20.1)**	**18.2 (5.2)**	**8 (9.2)**	**9 (7.7)**	**22.5 (5.1)**	**26.9 (12.4)**

Values are mean (standard deviation).

**Figure 1:**
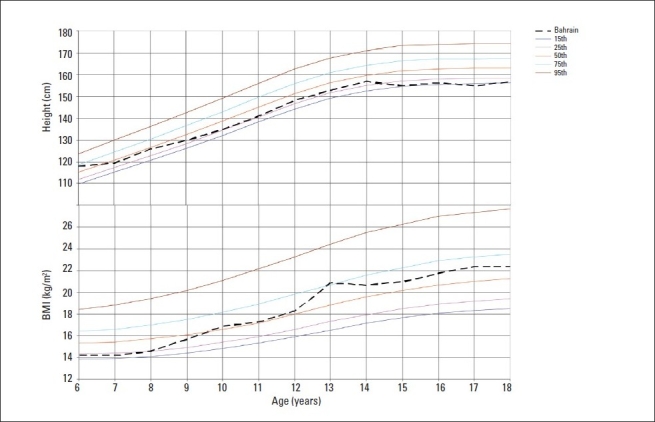
Median heights and BMI by age for girls compared to WHO growth reference for 2007 (15th, 25th, 50th, 95th percentiles).^14^

**Figure 2:**
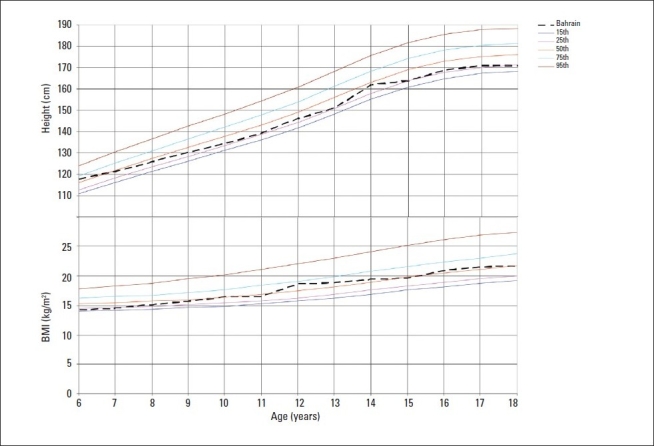
Median heights and BMI by age for boys compared to WHO growth reference for 2007 (15th, 25th, 50th, 75th, 95th percentiles).^14^

Figures 1 and 2 show the median heights and BMI of the study subjects plotted on WHO smoothed percentile charts.^14^ The median heights of Bahraini boys and girls aged 6 to 7 years were close to the 50th percentile of the WHO standards or higher; thereafter, median heights were closer to the 25th percentile up to age 18 years in boys and 13 years in girls. In late adolescence, the median height of girls showed a decline to the 15th percentile. The median BMI remained close to the WHO 50th percentile at all ages in boys and up to age 12 years in girls. In girls, BMI increased to between the 50th and 75th percentile during adolescence.

**Figure 3:**
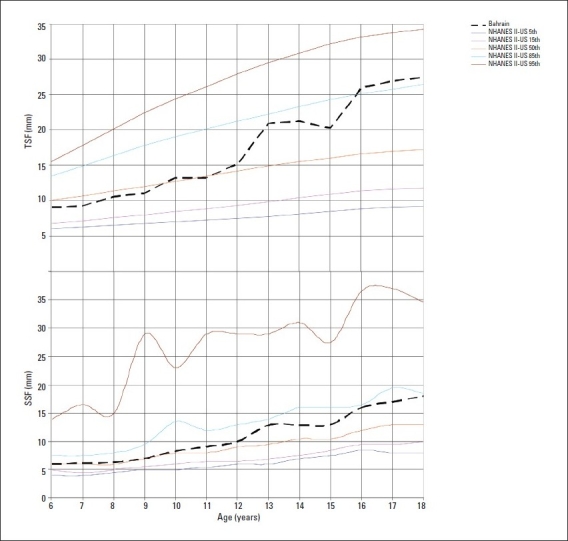
Median triceps skin fold thickness (TSF) and subscapular skin fold (SSF) thickness by age in girls compared to NHANES IIUS (5th, 15th, 50th, 85th, 95th percentiles).^16^

**Figure 4:**
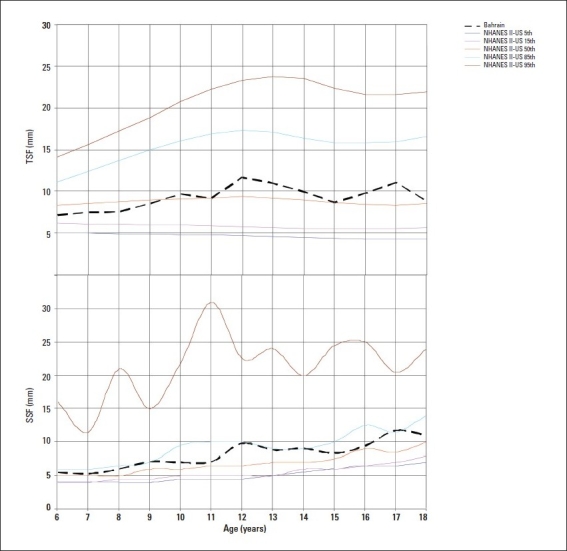
Median triceps skin fold thickness (TSF) and subscapular skin fold (SSF) thickness by age in boys compared to NHANES IIUS (5th, 15th, 50th, 85th, 95th percentiles).^16^

**Figure 5:**
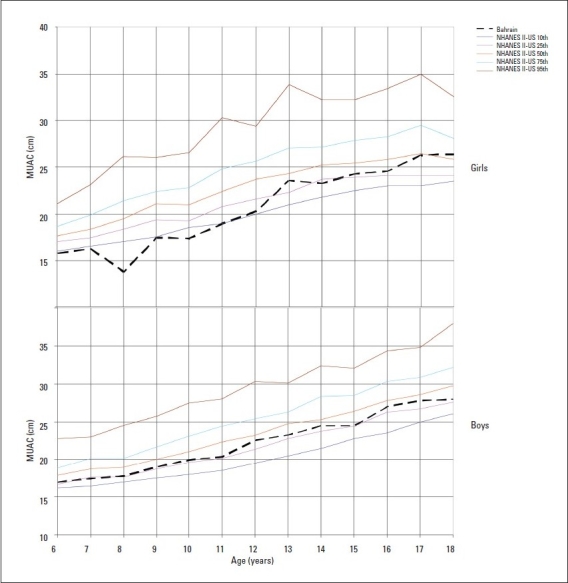
Median mid-upper arm circumference (MUAC) for boys and girls compared to NHANES II US standards (10th, 25th, 50th, 75th, 95th percentiles).^15^

The median values of TSF and SSF indicating subcutaneous fat, were generally close to the US 50th percentile during pre-adolescent years in both genders but increased thereafter to lie between the 75th and 85th percentiles (Figures 3 and 4). The median MUAC values plotted on NHANES-II-US^15^ smoothed percentiles were close to the 5th percentiles of the US standards for preadolescent girls, but increased thereafter reaching the 50th percentile at the age of 17 years. In boys, the values for MUAC remained close to the 25th percentile of the US standards at all ages (Figure 5). The median AMA of girls fluctuated between the 5th and 25th US percentile while that in boys remained close to the 25th percentile at all ages.

Tables 3 and 4 show that the Bahraini median percentile values for height of boys and girls were in general, lower than those of WHO at the 5th, 50th and 95th percentiles especially during adolescence when values were 3 to 6 cm lower than the reference standards. On the other hand, BMI values for Bahraini adolescents were higher by 3 to 6 kg/m^2^ at the 85th and 95th percentiles compared to WHO values.

**Table 3:** Distribution of percentiles of height among Bahraini girls and boys aged 6-18 years (2000-2001) compared to Who growth reference for 2007.^14^

Age (y)	Height Percentiles (cm)									
	Girls					Boys				
	Total	n	5	50	95	Total	n	5	50	95
6	51	50	110.6 (106.7)	118 (115.1)	132.5 (123.5)	36	31	111.9 (107.8)	118 (116)	130.6 (124.1)
7	89	87	110.6 (111.8)	119.5 (120.8)	130.9 (129.8)	100	89	113.8 (113)	121.5 (121.7)	133 (130.4)
8	96	90	114.6 (117)	126 (126.6)	133.2 (136.1)	116	113	115.9 (118)	126 (127.3)	135 (136.6)
9	126	119	119 (122.4)	130 (132.5)	145 (142.5)	100	98	120.5 (122.7)	130.5 (132.6)	142 (142.5)
10	120	116	125 (128.1)	135 (138.6)	151.1 (149.2)	106	101	120.2 (127.3)	134.5 (137.8)	145.9 (148.3)
11	131	123	130 (134.1)	141 (145)	153.5 (155.9)	114	110	130 (132)	139.5 (143.1)	151.7 (154.2)
12	111	104	134.6 (140)	148.3 (151.2)	159 (162.5)	90	90	132.3 (137.4)	146.3 (149.1)	166.5 (160.7)
13	93	83	139.7 (145)	153 (156.4)	162.9 (167.8)	108	106	136 (143.8)	151.3 (156)	165.3 (168.3)
14	105	97	145.9 (148.4)	157 (159.8)	168 (171.2)	113	98	144.5 (150.5)	162 (163.2)	174 (175.8)
15	84	71	147.5 (150.4)	155 (161.7)	163.6 (173)	93	85	146.6 (156.1)	154 (169)	178.4 (181.8)
16	112	108	147 (151.4)	156.5 (162.5)	168 (173.7)	94	86	155.4 (160.1)	169 (172.9)	181.3 (185.7)
17	106	97	147 (151.8)	155 (162.9)	168.6 (173.9)	98	90	160.6 (162.6)	171 (175.2)	180.7 (187.7)
18	96	90	146.6 (152.2)	157 (163.1)	167.5 (173.9)	82	76	160.4 (163.9)	171 (176.1)	182.2 (188.4)
**Total**	**1320**	**1235**				**1250**	**1173**			

WHO percentiles are in parentheses.

**Table 4:** Distribution of percentiles of body mass index among Bahraini girls and boys aged 6-18 years (2000-2001) compared to WHO growth reference for 2007.^14^

Age (y)	BMI Percentiles (kg/m^2^)											
	Girls						Boys					
	Total	n	5	50	85	95	Total	n	5	50	85	95
6	51	50	12.2 (13.1)	14.2 (15.3)	17.1 (17.1)	20.6 (18.4)	36	31	12.3 (13.4)	14.3 (15.3)	18.1 (16.8)	22 (17.9)
7	89	87	11 (13.1)	14.2 (15.4)	17.4 (17.4)	20.8 (18.8)	100	89	12.4 (13.5)	14.6 (15.5)	17.7 (17.1)	21.2 (18.3)
8	96	90	10.9 (13.3)	14.6 (15.7)	18.5 (17.8)	23.1 (19.4)	116	113	12.1 (13.7)	15.2 (15.7)	17.4 (17.5)	21 (18.8)
9	126	119	12.2 (13.6)	15.7 (16.1)	19.7 (18.4)	23.6 (20.2)	100	98	12.9 (13.9)	15.8 (16)	21.4 (18)	24.6 (19.5)
10	120	116	13.4 (13.9)	16.9 (16.6)	22.2 (19.1)	26.4 (21.1)	106	101	13.2 (14.1)	16.5 (16.4)	21.6 (18.6)	25.8 (20.2)
11	131	123	13.2 (14.4)	17.3 (17.2)	23.8 (20)	29.1 (22.2)	114	110	13.9 (14.5)	16.6 (16.9)	21.3 (19.3)	25.1 (21.1)
12	111	104	14.9 (14.9)	18.3 (18)	25.4 (20.9)	29.4 (23.3)	90	90	14.6 (14.9)	18.7 (17.5)	24.9 (20.1)	33.6 (22.1)
13	93	83	15.9 (15.5)	20.9 (18.8)	25.3 (21.9)	32 (24.4)	108	106	14.8 (15.4)	18.9 (18.2)	22.8 (20.9)	28.6 (23.1)
14	105	97	16.1 (16)	20.7 (19.6)	27.6 (22.9)	30.7 (25.5)	113	98	15.3 (16)	19.5 (19)	24.8 (21.9)	28.6 (24.2)
15	84	71	16.2 (16.5)	21 (20.2)	29.4 (23.7)	31.8 (26.3)	93	85	15.1 (16.5)	19.7 (19.8)	25.6 (22.8)	30.6 (25.2)
16	112	108	17.3 (16.8)	21.8 (20.7)	27.6 (24.2)	34.9 (27)	94	86	16.8 (17.1)	21 (20.5)	27.3 (23.7)	34.2 (26.1)
17	106	97	17.1 (17)	22.4 (21)	27.9 (24.7)	34.4 (27.4)	98	90	16 (17.5)	21.6 (21.1)	31.2 (24.4)	40.2 (26.9)
18	96	90	16.9 (17.1)	22.4 (21.3)	28.9 (24.9)	30.8 (27.7)	82	76	18.1 (17.9)	21.8 (21.7)	25.8 (25)	29.3 (27.5)
**Total**	**1320**	**1235**					**1250**	**1173**				

WHO percentiles are in parentheses.

## DISCUSSION

Anthropometric data are widely used to estimate the nutritional status of children. The height and weight of a child are useful indices of development, reflecting the various influences on growth, including nutrition. Indeed, the monitoring of a child's increase in height and weight by age using growth charts is widely used to identify failure-to-thrive or over nutrition.^10^,^18^

Among the scant number of published studies on anthropometric characteristics of Bahraini school children, only a few^2^,^3^ have reported data on a wide range of age categories of both genders, such as those in the present study. Moreover, compared to other studies^14^–^16^ the current research provides a much more comprehensive assessment of children's physical growth through the use of a variety of anthropometric indicators. Though the age of 6 years was less well represented than others due to few children of this age enrolled in grade 1 (36.4% girls and 26.5% boys), the number of children ≥7 years old were sufficient in each age group to estimate the standard deviation and percentiles with good precision. In general, the current study population could be considered representative of Bahraini school children.

Current data shows that Bahraini children are shorter at all ages compared to their NCHS/WHO reference counterparts except for the age group of 6 to 7 years old. This suggests that children had a good growth phase during the preschool age period and excludes the probability of severe or prolonged malnutrition in the critical growth periods of intrauterine life, infancy and the preschool ages. Similar results were reported by Rasheed et al^6^ in their study on Saudi children.

Compared to findings of an earlier study,^3^ the growth performance of Bahraini children has changed. Median heights of Bahraini children two decades ago fell between the 5th and 25th percentiles of the NCHS/ US standards, while median weights were below the 50th percentiles of the standard. Current findings suggest that Bahraini children are heavier and slightly taller than their counterparts 10 to 15 years ago. These secular changes in the growth patterns of children are probably a reflection of better living conditions in Bahrain in recent years. Data on secular trends for growth patterns in certain developed and developing countries have also shown a marked intergenerational increase in body size and a tendency toward earlier sexual maturation as the socioeconomic conditions and nutritional status of populations have improved.^19^

Earlier studies from other Arab Gulf states such as those from Oman and Saudi Arabia^4^–^6^,^20^ also showed that children in these countries were shorter and lighter than their counterparts in the present study. However, a recent study by El Mouzan et al for the development of reference growth charts of Saudi children and adolescents showed comparable findings for height and weight.^21^ The current findings of low height-for-age and normal/high weight-for-height in boys and girls compared to the reference population suggest a genetic control on height. On the other hand, studies are now reporting the possibility of an environmental control. Becker and his colleagues propose that the important association between stunting and high weight-for-height in a variety of ethnic, environmental and social backgrounds is possibly a consequence of nutritional insults during pregnancy and infancy that have long-term effects on a wide range of metabolic and other relationships.^22^ Although the underlying mechanisms remain unexplained, this biological phenomenon could raise public health concerns in countries experiencing nutrition transition and changes in activity pattern.

Compared to NCHS/WHO reference standards the distribution of BMI was positively skewed for Bahraini girls during adolescence, indicating a trend toward overnutrition in this age group. These observations are in line with the high BMI distributions seen among Bahraini women (≥19 years) in a recent national nutrition survey (2000).^23^ Flegal and Troiano^24^ in the US suggested that the causes for widespread obesity lie mainly at the population level with social and environmental factors playing an important role. Better socioeconomic circumstances in transitional societies generally leads to increased availability of food, changes in food composition and patterns of food intake, less time spent on physical activity and perhaps also changes in cultural and social attitudes and values that directly or indirectly affect body weight. All these factors exist in this region and might explain, to a large extent, the high prevalence of overweight and obesity.^23^,^25^

Current findings also show that the BMI cut-off value for overweight status (85th percentile) is higher than that of the WHO reference especially during adolescence. Hence, use of the WHO charts is likely to show exaggerated prevalence estimates of overweight/obesity in Bahraini children. We suggest that local reference standards be developed for Bahraini children and adolescents.

Compared to findings of earlier studies from Bahrain and Saudi Arabia,^3^,^4^ the current generation of Bahraini children show substantially higher values in arm circumference size. Assessment of mean AMA values of current Bahraini adolescents were close to observations made by a recent local study,^26^ but lower than those reported for American and German boys and girls of corresponding ages.^16^,^27^ This variation in muscularity with Western children may be due to differences in genetic as well as environmental factors such as level of physical activity related to use of arms and/or variations in dietary patterns. A lower AMA among girls than boys is perhaps a consequence of greater sedentarism among girls of this region as well as physiological causes.^28^

Measurement of skin fold thicknesses, as a specific index for obesity, has some advantage over the weight-for-height.^29^ While high weight-for height may be due to excessive muscularity as found in athletes, skin fold thickness provides an indirect estimate of total body fat. Current TSF thickness findings indicate a higher accumulation of body fat in the arms of Bahraini vs. US children. Median TSF thickness Bahraini values were also higher than those of children from the UK (7-10 years of age),^30^ Germany (6-18 years of age),^27^ and Mexico (5-9.9 and 10-17.9 years of age).^28^ Compared to findings of surveys conducted in this region a decade ago^3^,^4^ current values for skin-fold thicknesses (TSF and SSF) were much higher, indicating the rising secular trend of overnutrition. A recent study^1^ reported higher values for TSF thickness in Bahraini adolescents than found in our study. This difference is probably due to variation in the type of skin-fold measuring calipers used. Whereas we used the Harpenden calipers, Al-Sendi et al1 used the Holtain and Lange calipers. Variation in measurements have been reported in individuals with the use of different types of calipers.^18^

Median SSF thickness values in Bahraini children were also higher than those of American children (NHANES-II) during adolescence. Both total body fat and regional fat deposition in childhood or adolescent obesity have been associated with adult disease.^31^ Researchers have pointed that children with both high BMI and trunk skin fold values have an increased risk of centralized obesity in adult age, which in turn has an increased risk of cardiovascular disease.^32^ Moreover, centralized or upper body fat in children carries an increased risk for metabolic complications, such as increased levels of plasma low-density lipoprotein cholesterol, triglycerides, basal insulin and low levels of high density lipoprotein cholesterol.^33^–^35^

The findings of the present study on BMI as well as on TSF and SSF thickness suggest an increased trend for adiposity among Bahraini adolescents, especially in girls, which puts this age group at a higher risk of adult obesity and its consequences. A need for urgent intervention programs is emphasized.
